# Chamaejasmine Arrests Cell Cycle, Induces Apoptosis and Inhibits Nuclear NF-κB Translocation in the Human Breast Cancer Cell Line MDA-MB-231

**DOI:** 10.3390/molecules18010845

**Published:** 2013-01-11

**Authors:** Tingting Zhang, Hongyang Yu, Guanglu Dong, Li Cai, Yuxian Bai

**Affiliations:** 1 Oncology Department of Internal Medicine, the Third Affiliated Hospital of Harbin Medical University, Harbin 150040, China; E-Mail: zhangting2002@sina.com; 2 Department of Radiation Oncology, the Second Affiliated Hospital of Harbin Medical University, Harbin 150086, China; E-Mails: 18704665791@139.com (H.Y.); dgl64@163.com (G.D.)

**Keywords:** chamaejasmine, MDA-MB-231, MTT, flow cytometry, western blotting

## Abstract

In this study, the anticancer activity of chamaejasmine was characterized in the human breast cancer cell line, MDA-MB-231. Cell viability and cell cycle distribution were determined by MTT assay and flow cytometry, respectively. Western blotting was performed to determine changes in levels of various proteins. Results showed that treatment with chamaejasmine (4–16 μM) inhibited cell proliferation, which correlated with G2/M phase arrest and apoptosis in MDA-MB-231 cells. Chamaejasmine treatment of MDA-MB-231 cells resulted in induction of WAF1/p21 and KIP1/p27, decrease in cyclins A and cyclins B1. Cyclin-dependent kinase (cdk) 2 and cdc2 was also decreased after chamaejasmine treatment. Moreover, inhibition of nuclear translocation, phosphorylation of NF-κB, activation of IKKα and IKKβ, inhibition of phosphorylation and degradation of IκBα were also detected in this work. Our findings suggested that chamaejasmine could be explored as a preventive and perhaps as a chemotherapeutic agent in the management of breast cancer.

## 1. Introduction

Breast cancer, a major malignant tumor threatens women’s health, and is the second leading cause of women’s death [1]. It threatens women in all races [2]. Surgery and chemotherapy or/and radiation are usually used for breast cancer treatment [3,4]. Therapies such as paclitaxel (taxol) are commonly used to treat breast cancer, but these drugs usually have frightening toxicity to normal cells [5]. Therefore, an understanding of the molecular mechanisms of more effective and less harmful potential medicines is sorely needed.

Cell cycle control is a highly regulated process in which cyclin-dependent kinases, cyclin-dependent kinase inhibitors, and cyclins play essential roles [6]. The regulation of the expression and function of the cell cycle machinery proteins (including cyclins, CDKs, CKIs, p21, and p27) has provided an important mechanism for inhibiting cell growth [7].

Apoptosis, a form of programmed cell death, occurs through activation of the cell’s intrinsic suicide machinery [8]. It is an important hallmark of anticancer drug-induced cell death [9,10,11]. Apoptosis is characterized by condensation of cytoplasm and chromatin, DNA fragmentation, and cell fragmentation into apoptotic bodies, followed by removal and degradation of the dying cells by phagocytosis. It has been considered as the major form of cell death in various physiological events [12,13].

**Figure 1:**
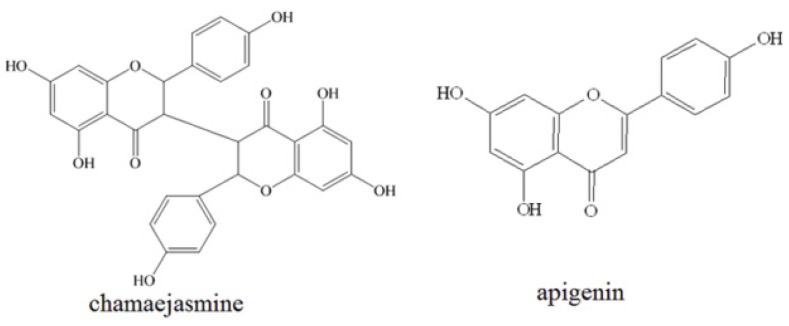
Chemical structure of chamaejasmine and apigenin.

Recently, numerous medical plants have served as sources of anticancer pharmaceuticals, and more than 60% of current anticancer drugs such as vinblastine, topotecan, etoposide, and paclitaxel were originally plant-derived compounds [14,15]. *Stellerachamaejasme* L. (Thymealaeaceae) is widely distributed in the northwest and southwest parts of China. It has been reported that the roots of *Stellerachamaejasme* L., could be used as a pesticide to kill bugs, flies and maggots, and could also control pests on crops, and pastures [16,17]. Moreover, the methanol extract of *Stellerachamaejasme* L. showed significant antitumor activities [18]. Chemical constituent investigations indicated *Stellerachamaejasme* L. is rich in biflavonones, which have been considered as being responsible for the beneficial effects of *Stellerachamaejasme* L. on human health [19]. Chamaejasmine (Figure 1), a natural biflavanone, was one of the major biflavanones obtained from *Stellerachamaejasme* L. [20]. As far as we know, the anticancer activity of chamaejasmine against MDA-MB-231 has not been elucidated until now.

In the present study, the antiproliferation activity of chamaejasmine against three human breast cancer cell lines (HCC1937, MDA-MB-453 and MDA-MB-231) was evaluated by MTT assay first. The cell cycle arrest and apoptosis was further studied by flow cytometry. The expression of p21, p27, cdk2, cdc2, cyclin A and cyclin B1 was further detected by western blotting in MDA-MB-231 cells. Measurements of Bcl-2, Bax, caspase-3 and capspase-8 were used to assess apoptosis. Finally, we determined the chemotherapeutic potential of chamaejasmine on phosphorylation and activation of NF-κB in MDA-MB-231 cells.

## 2. Results

### 2.1. Heading Cytotoxicity Assays

The cytotoxicity of chamaejasmine was evaluated on three human breast cancer cell lines (HCC1937, MDA-MB-453 and MDA-MB-231) using MTT assays. Apigenin was used as positive control. The results were listed in Table 1. Chamaejasmine exhibited stronger inhibition against all three cancer cell lines than apigenin. Among all of them, chamaejasmine showed more notable cytotoxicity against MDA-MB-231 than HCC1937 and MDA-MB-453, with IC_50_ values of 4.72, 13.44 and 5.66 μM, respectively.

**Table 1:** Inhibition concentrations 50% (IC_50_) values for chamaejasmine towards HCC1937, MDA-MB-453 and MDA-MB-231cells determined by MTT assay. * Statistically different from positive control (apigenin, *p* < 0.05).

Cell lines	IC_50_ (μM)	
	Chamaejasmine	Apigenin
HCC1937	13.44 *	35.97
MDA-MB-453	5.66 *	31.12
MDA-MB-231	4.72 *	21.77

### 2.2. G2-M Phase Cell Cycle Arrest and Apoptosis by Chamaejasmine in MDA-MB-231 Cells

**Figure 2:**
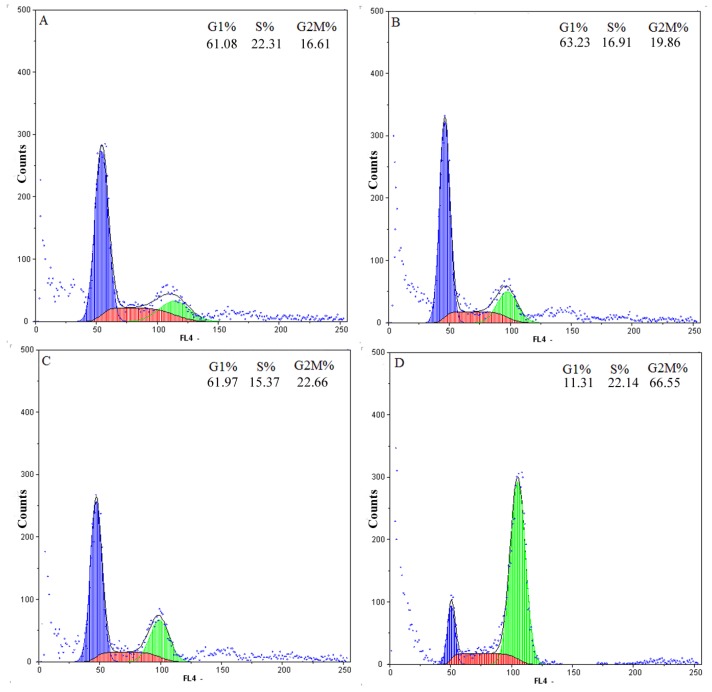
Cell cycle distribution of MDA-MB-231 cells after treatment with different concentrations of chamaejasmine for 48 h. (**A**), treatment with 0 μM chamaejasmine; (**B**), treatment with 4 μM chamaejasmine; (**C**), treatment with 8 μM chamaejasmine; (**D**), treatment with 16 μM chamaejasmine. Blue = G1; red = S; green = G2/M. The figure shown here are representative of three independent experiments with similar results.

To determine whether chamaejasmine-induced apoptosis was related to arrest of cell cycle progression, flow cytometry was used to quantitate the cell cycle distribution in MDA-MB-231 cells under treatment with different chamaejasmine concentrations (4–16 μM). As shown in the concentration kinetic measurements (Figure 2), exposure to 4–16 μM chamaejasmine caused an increase of the G2/M phase population from 19.86% to 66.55%, as compared to 16.61% of G2/M phase cells in untreated control samples (*p* < 0.05). Hence, chamaejasmine exerted growth-inhibitory effects via G2/M phase arrest in a concentration-dependent manner.

**Figure 3:**
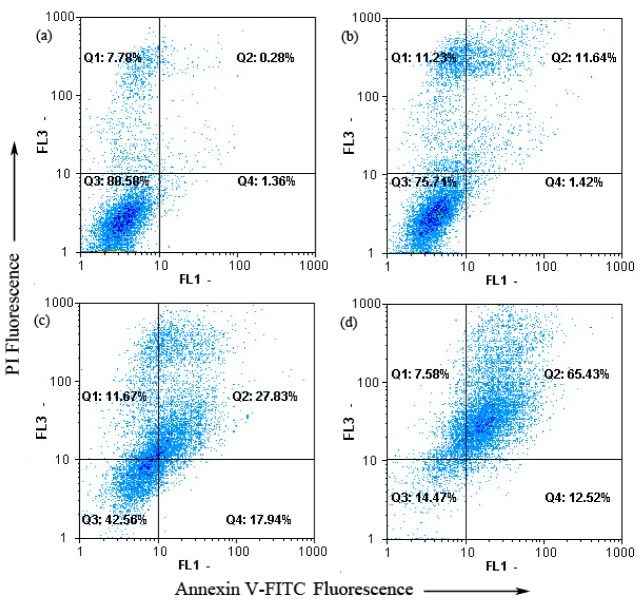
Chamaejasmine induced apoptosis in MDA-MB-231 cells using annexinV-FITC/PI. (**a**) Treatment with 0 μM chamaejasmine; (**b**) treatment with 4 μM chamaejasmine; (**c**) treatment with 8 μM chamaejasmine; (**d**) treatment with 16 μM chamaejasmine.

The annexin V-FITC apoptosis detection kit was then employed to examine the influence of chamaejasmine on MDA-MB-231 cells apoptosis by flow cytometry. As shown in Figure 3, only a few untreated MDA-MB-231 (1.64%) cells bounded annexin V-FITC. Whereas, MDA-MB-231 cells binded annexin V-FITC highly increased in a concentration-dependent manner after treatment with 4–16 μM chamaejasmine (13.06% to 78.05%, *p* < 0.05). To sum up, dots were dispersed and shifted to the Q2 side in a dose-dependent manner when MDA-MB-231 cells were treated with chamaejasmine, indicating that the cells moved to the late apoptotic stage. These experimental results demonstrate that chamaejasmine induced apoptosis of MDA-MB-231 cells.

### 2.3. Inhibition of Cyclins, Cdk2, cdc2 and Induction of WAF1/p21 and KIP1/p27 by Chamaejasmine in MDA-MB-231 Cells

**Figure 4:**
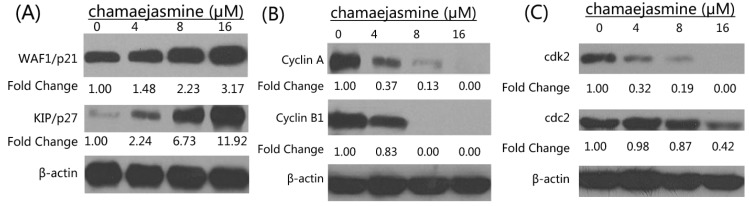
(**A**) Effects of chamaejasmine on protein expression of WAF1/p21 and KIP1/p27 in MDA-MB-231 cells. (**B**) Effects of chamaejasmine on protein expression of cyclin A and cyclin B1 in MDA-MB-231 cells. (**C**) Effects of chamaejasmine on protein expression of cdk2 and cdc2 in MDA-MB-231 cells. As detailed in Materials and Methods, the cells were treated with chamaejasmine (4–16 μM) and then harvested. Total cell lysates were prepared and 20 μg proteins were subjected to SDS-PAGE followed by western blotting analysis. Equal loading of proteins was confirmed by stripping the western blotting and reprobing it for β-actin. The western blotting shown here are representative of three independent experiments with similar results. The values below the figures represent relative density of the bands normalized to β-actin.

Many reports have revealed that cell cycle regulators are frequently mutated in most common malignancies [21,22]. Thus, we examined the effects of chamaejasmine on cell cycle inhibitory proteins KIP1/p27 and WAF1/p21, which are involved in cell cycle progression. Western blotting analysis showed a significant induction of these proteins in a dose-dependent manner (Figure 4A). The effects of chamaejasmine on the proteins levels of cyclins, cdk2 and cdc2 (which are known to be regulated by KIP1/p27 and WAF1/p21) were next evaluated. Chamaejasmine treatment of cells resulted in a significant dose-dependent decrease in the protein levels of cyclin A and B1 as well as cdk2 and cdc2 (Figure 4B,C). These results suggested that chamaejasmine restored proper checkpoint control via modulation of the cyclins, cdk2, cdc2 and the expression of their inhibitors.

### 2.4. Induction of Bax and Inhibition of Bcl-2 and Procaspases in MDA-MB-231 Cells

**Figure 5:**
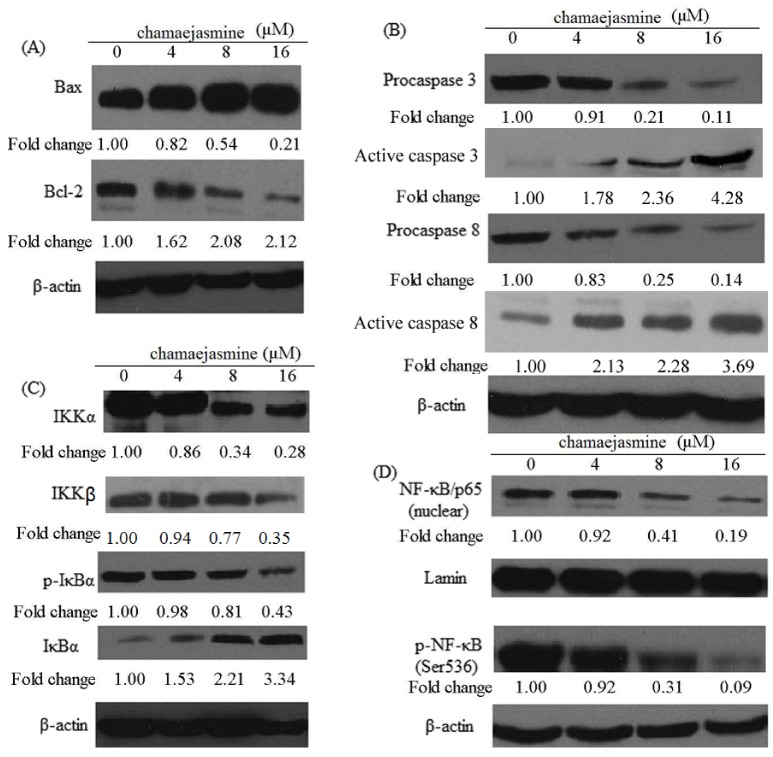
(**A**) Effects of chamaejasmine on proteins expression of Bax, Bcl-2 in MDA-MB-231cells. (**B**) Effects of chamaejasmine on proteins expression of caspase-3 and -8 in MDA-MB-231 cells. (**C**) Effects of chamaejasmine on IKKα, IKKβ, phosphorylation IKBα as well as degradation of IκBα in MDA-MB-231 cells. (**D**) Effects of chamaejasmine on activation of NF-κB in MDA-MB-231 cells by western blotting analysis. As detailed in Materials and Methods, the cells were treated with chamaejasmine (4–16 μM) and then harvested. Total cell lysates were prepared and 20 μg proteins were subjected to SDS-PAGEfollowed by western blotting analysis. Equal loading of proteins was confirmed by strippingthe western blot and reprobing it for β-actin. The western blots shown here are representativeof three independent experiments with similar results. The values below the figures represent relative density of the bands normalized to β-actin.

In order to investigate the mechanism by which chamaejasmine induces apoptosis, the changes in the level of apoptosis-related proteins (Bax, Bcl-2, caspase-3 and caspase-8) were examined (Figure 5A,B). As shown in Figure 5A, western blotting analysis revealed a significant increase in the expression of Bax in chamaejasmine treated MDA-MB-231 cells, while there was a significant decrease in Bcl-2 expression, indicating that the Bax/Bcl-2 ratio increased significantly. Pro-caspase-3 levels decreased upon treatment with chamaejasmine, while the levels of active caspase-3 increased. Similarly, pro-caspase-8 levels decreased upon treatment with chamaejasmine which induced increase active caspase-8 (Figure 5B).

### 2.5. Inhibition of NF-κB Pathway by chamaejasmine in MDA-MB-231 Cells

**Figure 6:**
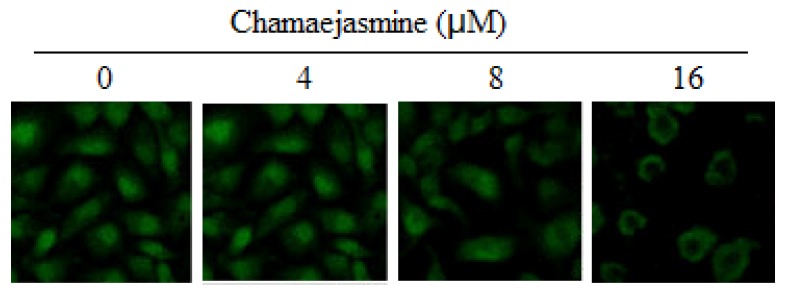
Immunofluorescence analysis of NF-κB (p65 subunit) localization in the nuclei of MDA-MB-231 cells after 0–16 μM chamaejasmine treatment. A repeat experiment gave very similar results.

Treatment of MDA-MB-231 cells with chamaejasmine (4–16 μM) resulted in a significant inhibition in the phosphorylation of IκBα protein (Figure 5C). To evaluate the possible inhibitory mechanism of chamaejasmine on IκBα protein increase, IKKα and IKKβ protein level was then measured. Western blotting analysis showed that pre-treatment of MDA-MB-231 cells with chamaejasmine inhibited IKKα and IKKβ in a dose-dependent manner (Figure 5C). Furthermore, chamaejasmine treatment cells resulted in decreased phospho-NF-κB/p65 at Ser536 and inhibition of translocation of NF-κB/p65 in the nuclear fraction (Figure 5D and Figure 6). 

## 3. Discussion

Recently, the antitumor activity of constituents obtained from *Stellerachamaejasme* L. has become a research hotspot. For example, chamaejasmine showed notable anticancer activity against HEp-2, NCI-H1975, HT-29 and SKOV-3. MCF-7, A549, SGC-7901, HCT-8, HO-4980, Hela, HepG2, PC-3, LNCap, with the IC_50_ values of 1.92, 3.61, 14.30, 10.67, 8.04, 4.02, 4.84, 11.97, 12.45, 5.31, 9.88, 14.36, 2.28 and 5.21 μM [23,24]. Furthermore, daphnoretin was found to obviously inhibit HOS, U2-OS and MG-63, with the IC_50_ values of 3.89, 8.72 and 5.11 µM [25]. Besides, chamaejasmine exhibited strong inhibition against all three human breast cancer cell lines (HCC1937, MDA-MB-453 and MDA-MB-231). Based on all above, it seemed that the presence of hydroxyl group may contribute to the obvious anticancer activity of chamaejasmine. The antitumor activity of biflavanones or their structural derivatives need to be further evaluated in cancer cell lines.

Several studies have shown that the induction of apoptosis might be due to cell cycle arrest [26,27]. Cell cycle control is a major regulatory mechanism of cell growth [28]. Blockade of the cell cycle is considered as an effective strategy for the development of novel cancer therapies [29,30]. Cell cycle analysis of the treated culture revealed that chamaejasmine induced a concentration-dependent G2/M phase cell cycle arrest with an accompaniment decrease in G1 and S phase.

It is known that cell cycle is primarily regulated by complexes containing cdks and cyclins, which are critical for the progression of cell cycle and whose inactivation leads to cell cycle arrest [31,32]. Cdk activity is additionally regulated by cdk inhibitors such as the WAF1/p21 and KIP1/p27 proteins families. Among Cdks that regulate cell cycle progression, Cdk2 and Cdc2 kinases are primarily activated in association with cyclin A and cyclin B1 during the progression of the G2/M phase [33,34]. Thus, our data suggest that cell cycle arrest at the G2/M phase is mediated by reduction of cyclin A and cdc2/cyclin B complex formation, which is an essential step in regulating the cells passage into mitosis. It could be conclude the cell cycle arrest may partly explain apoptosis and anti-proliferative effects induced by chamaejasmine.

Bcl-2, an upstream effect or molecule in the apoptotic pathway, has been identified as a potent suppressor of apoptosis [35]. It has been proven that most cancers, including lung cancer, generally overexpress Bcl-2, thereby escaping apoptosis and undermining therapy. Bcl-2 forms a heterodimer with the apoptotic protein Bax and thereby neutralizes its apoptotic effect. Therefore, alteration in the ratio of Bax/Bcl2 is a decisive factor that plays an important role to determine whether cells will undergo apoptosis [36,37]. Based on the above results, chamaejasmine significantly down-regulated Bcl-2 protein and up-regulated levels of Bax protein in MDA-MB-231 cells, suggesting the involvement of an intrinsic apoptotic pathway by which chamaejasmine induces apoptosis in MDA-MB-231 cells.

Caspases are part of a growing family of cysteine proteases which have been involved in many forms of apoptosis [38,39,40]. Activation of caspase proteases was required for the induction of apoptosis in different cell types [41,42]. Caspases include initiator caspases and effector caspases. Once the initiator caspases (caspases 8, 9, and 10, *etc*) are activated through intrinsic or extrinsic pathway, they are proteolytically cleaved and thus activate the effector caspases (caspases 3, 6, and 7, *etc*.) whose functions are known to be responsible for the cleavage of the intracellular substrates that leads to cell death. Caspase 3 is one of the key executioners of apoptosis. Upon activation, caspase 3 can cleave 5 substrates, including other effector caspases and fodrin, which form a cytoskeletal network [43]. The activated caspase-3 and caspase-8 detected in the results further explained the signaling pathway of chamaejasmine-induced apoptosis in MDA-MB-231 cells.

Activation of NF-κB has been confirmed to block apoptosis and promote cell proliferation, and its increased activity is positively associated with many cancer types, including breast cancer [44]. Inhibition of tumorigenesis often involves modulation of signal transduction pathways, leading to cell cycle arrest and apoptosis. NF-κB is sequestered in the cytoplasm in an inactive form through interaction with IκB. Phosphorylation of IκB by IκB kinase (IKK) causes ubiquitination and degradation of IκB, thus releasing NF-κB, which then translocates to the nucleus. Then it binds to specific B binding sites in the promoter regions of several genes [45]. Our results showed that treatment of chamaejasmine in MDA-MB-231 cells significantly inhibited IKKα and IKKβ as well as phosphorylation and degradation of IκBα protein. This suggested that the effects of chamaejasmine on NF-κB/p65 are through inhibition of phosphorylation and subsequent proteolysis of IκBα. NF-κB is a ubiquitous transcription factor that controls the expression of genes involved in immune responses, apoptosis, and cell cycle. It has been shown to modulate the expression of cyclin B1 [46,47]. Moreover, NF-κB plays important role in p53-mediated apoptosis. P53 can induce activation of NF-kB, and loss of NF-kB activity specially abrogated the p53-mediated apoptotic response, without impinging on the ability to activate expression of target genes or induce cell-cycle arrest [48].

## 4. Experimental

### 4.1. Materials

Bax, Bcl-2, IκBα, IκBα (phospho), anticyclinA, B1, cdk2, cdc2, WAF1/p21and KIP1/p27, *p*-NF-κB/p65 antibodies were obtained from Cell Signaling Technology (Beverly, MA, USA). The mono and polyclonal antibodies IKKα, IKKβ, Procaspase 3, 8 and NF-κB/p65 were obtained from Santa Cruz Biotechnology Inc. (Santa Cruz, CA, USA). Anti-mouse and anti-rabbit secondary antibody alkaline phosphatase peroxidase conjugate was obtained from Amersham Life Science Inc. (Arlington Height, IL, USA). BCA Protein assay kit was obtained from Pierce (Rockford, IL, USA). Novex precast Tris-glycine gels were obtained from Invitrogen (Carlsbad, CA, USA). The Apo-Direct kit for flow cytometry was purchased from Phoenix Flow Systems (San Diego, CA, USA).

### 4.2. Cell Culture and Chemicals

Three human breast cancer cell lines (HCC1937, MDA-MB-453 and MDA-MB-231) were obtained from The Cell Bank of Type Culture Collection of Chinese Academy of Sciences, Shanghai Institute of Cell Biology, Chinese Academy of Sciences (Shanghai, China). HCC1937 was maintained in RPMI-1640 (Invitrogen Life Technologies, Carlsbad, CA, USA), and MDA-MB-453 and MDA-MB-231 was maintained in L-15 (Invitrogen Life Technologies, Carlsbad, CA, USA). The medium were supplemented with 10% fetal bovine serum (FBS, Gibco, Carlsbad, CA, USA), 100 U/mL penicillin and 100 μg/mL streptomycin at 37 °C and 5% CO_2_. Chamaejasmine (purity ≥ 99%, molecular weight: 542.49) were obtained from National Research Center for Certified Reference Materials (Beijing, China). Apigenin (purity ≥ 99%, molecular weight: 270.25) were obtained from Sigma Chemical Co. (St. Louis, MO, USA). A 10 mM stock solution of chamaejasmine was prepared in dimethyl sulfoxide (DMSO) and stored at −80 °C. MTT was also obtained from Sigma-Aldrich Inc. Deionized water was used in all experiments.

### 4.3. Cytotoxicity Assay

Inhibition of cell proliferation of chamaejasmine was measured by the MTT assay [49]. Briefly, cells were plated in 96-well culture plates (1 × 10^5^ cells/well) separately. After 24 h incubation, cells were treated with chamaejasmine or apigenin (1, 2, 4, 8, 16, 32 and 64 μM, eight wells per concentration) for 72 h, MTT solution (5 mg/mL) was then added to each well. After 4 h incubation, the formazan precipitate was dissolved in dimethyl sulfoxide (100 μL) and then the absorbance was measured in an ELISA reader (Thermo Molecular Devices Co., Union City, CA, USA) at 570 nm. The cell viability ratio was calculated by the following formula: Inhibitory ratio (%) = [(OD_control_ − OD_treated_)/(OD_control_)] × 100%. Cytotoxicity was expressed as the concentration of chamaejasmine inhibiting cell growth by 50% (IC_50_ value).

### 4.4. Flow Cytometric Analysis of Cell Cycle and Apoptosis

Cell cycle was studied with CyStain (Partec GmbH, Görlitz, Germany) [49]. Briefly, 1 × 10^6^ cells/well MDA-MB-231 cells were seeded in six-well plate and left for 24 h in incubator to resume exponential growth. Cells were exposed to chamaejasmine (0, 4, 8 and 16 μM) and incubated for 48 h. Then, the cells were harvested and washed with PBS. After suspension in PBS (800 μL) and CyStain (200 μL) the cell cycle distribution of 10,000 cells was recorded by flow cytometry (Partec), and the percentage of cells at G0/G1, S, and G2/M phases was analyzed using the FloMax software (Partec).

The extent of apoptosis was measured through annexinV-FITC apoptosis detection kit (Beyotime Institute of Biotechnology, Jiangsu, China) as described by the manufacture’s instruction [49]. After exposure to chamaejasmine (0, 4, 8 and 16 μM) for 48 h, cells were collected, washed twice with PBS, gently resuspended in annexinV binding buffer and incubated with annexinV-FITC/PI in dark for 15 min and analyzed by flow cytometry using FloMax software. The fraction of cell population in different quadrants was analyzed using quadrant statistics. The lower left quadrant contained intact cells; lower right quadrant apoptotic and in the upper right quadrant necrotic or post-apoptotic cells.

### 4.5. Preparation of Cytosolic and Nuclear Lysates

Following treatment of cells with chamaejasmine (0, 4, 8 and 16 μM, 48 h), the medium was aspirated, and the cells were washed twice with PBS (10 mM, pH 7.4). The cells were incubated in 0.2 mL ice-cold lysis buffer (HEPES (10 mM, pH 7.9), KCl (10 mM), EDTA (0.1 mM), EGTA (0.1 mM), DTT (1 mM), PMSF (1 mM) with freshly added protease inhibitor cocktail (Protease Inhibitor Cocktail Set III; Calbiochem, La Jolla, CA, USA) for 15 min, after which 12.5 μL of 10% Nonidet P-40 was added and the contents were mixed on a vortex and then centrifuged for 1 min (14,000 *g*) at 4 °C. The supernatant was saved as cytosolic lysate and stored at −80 °C. The nuclear pellet was resuspended in 50 μL of ice-cold nuclear extraction buffer [HEPES (20 mM, pH 7.9), NaCl (0.4 M), EDTA (1 mM), EGTA (1 mM), DTT (1 mM), PMSF (1 mM)] with freshly added protease inhibitor cocktail (Protease Inhibitor Cocktail Set III; Calbiochem) for 30 min with intermittent mixing. The tubes were centrifuged for 5 min (14,000 g) at 4 °C, and the supernatant (nuclear extract) was stored at −80 °C.

### 4.6. Western Blotting Assay

To evaluate the expression levels of various intracellular proteins related to apoptosis, MDA-MB-231 cells were treated with chamaejasmine (0, 4, 8 and 16 μM) for 48 h, respectively. For isolation of total protein fractions, cells were collected, washed twice with ice-cold PBS, and lysed using cell lysis buffer [20 mMTris pH 7.5, 150 mMNaCl, 1% Triton X-100, 2.5 mM sodium pyrophosphate, 1 mM EDTA, 1% Na2CO3, 0.5 μg/mL leupeptin, 1 mM phenylmethylsulfonyl fluoride (PMSF)]. The lysates were collected by scraping from the plates and then centrifuged at 10,000 rpm at 4 °C for 5 min. Total protein samples (20 μg) were loaded on a 12% of SDS polyacrylamide gel for electrophoresis, and transferred onto PVDF transfer membranes (Millipore, Billerica, MA, USA) at 0.8 mA/cm^2^ for 2 h. Membranes were blocked at room temperature for 2 h with blocking solution (1% BSA in PBS plus 0.05% Tween-20). Membranes were incubated overnight at 4 °C with primary antibodies at a 1:1,000 dilution in blocking solution. After thrice washings in TBST for each 5 min, membranes were incubated for 1 h at room temperature with an alkaline phosphatase peroxidase conjugated anti-mouse secondary antibody at a dilution of 1:500 in blocking solution. Detection was performed by the BCIP/NBT Alkaline Phosphatase Color Development Kit (Beyotime Institute of Biotechnology) according to the manufacturer’s instructions. Bands were recorded by a digital camera (Nikon, Tokyo, Japan).

### 4.7. Immunofluorescence Assay

The cells were grown at a density of 2 × 10^4^/well on 6-well culture plates (Nunc Inc., Naperville, IL, USA). After the treatments, the cells were washed with PBS and fixed with cold methanol for 5 min at −20 °C. The cells were incubated with a 1:100 dilution of anti-p65 antibody, followed by probing with a 1:800 dilution of Alexa Fluor 488—conjugated goat anti-rabbit IgG. The cells were examined with a Zeiss Axiovert 200M microscope, and data were analyzed using Carl Zeiss Axiovision software (Carl Zeiss Instruments, Jena, Germany).

### 4.8. Statistical Analysis

The data were expressed as mean ± S.D. All statistics were calculated using the STATISTICA program (StatSoft, Tulsa, OK, USA). A *p*-value of <0.05 was considered as significant.

## 5. Conclusions

In summary, the present study showed that chamaejasmine inhibited the growth of MDA-MB-231 cells resulted from cell cycle arrest at G2/M phase, accompanied by cell apoptosis. Chamaejasmine induced cell cycle arrest through cyclinA, cyclinB1, cdk2 and cdc2 inhibition. These events were found to be associated with alterations in the levels of Bax, Bcl-2, caspase-8 and caspase-3. Our results further showed that the effects of chamaejasmine on NF-κB/p65 are through inhibition of phosphorylation and subsequent proteolysis of IκBα. All these evidences provide a rationale to explore chamaejasmine as a preventive and perhaps as a chemotherapeutic agent in the management of breast cancer.
